# Potential Impact of Carry-Over Effects in the Dynamics and Management of Seasonal Populations

**DOI:** 10.1371/journal.pone.0155579

**Published:** 2016-05-12

**Authors:** Eduardo Liz, Alfonso Ruiz-Herrera

**Affiliations:** Departamento de Matemática Aplicada II, Universidad de Vigo, 36310 Vigo, Spain; University of Alberta, CANADA

## Abstract

For many species living in changing environments, processes during one season influence vital rates in a subsequent season in the same annual cycle. The interplay between these carry-over effects between seasons and other density-dependent events can have a strong influence on population size and variability. We carry out a theoretical study of a discrete semelparous population model with an annual cycle divided into a breeding and a non-breeding season; the model assumes carry-over effects coming from the non-breeding period and affecting breeding performance through a density-dependent adjustment of the growth rate parameter. We analyze the influence of carry-over effects on population size, focusing on two important aspects: compensatory mortality and population variability. To understand the potential consequences of carry-over effects for management, we have introduced constant effort harvesting in the model. Our results show that carry-over effects may induce dramatic changes in population stability as harvesting pressure is increased, but these changes strongly depend on whether harvesting occurs prior to reproduction or after it.

## Introduction

Population dynamics of many species are influenced by seasonality, and seasonal interactions have the potential to modify important factors such as population abundance [1, 2], population stability [3, 4], and persistence [5, 6].

Models of population dynamics explicitly involving seasonality illustrate how density dependence can lead to seasonal compensation effects, which occur when a change in population size in a season affects the strength of density dependence (and hence the reproductive success) in the following season [1, 7, 8]. Another type of seasonal interaction is described by carry-over effects that occur at the individual level in one season and influence individual success in the following season [7]. The difference between carry-over effects and seasonal density dependence is the level at which they are defined (individual level versus population level) [2].

Since there is empirical evidence that carry-over effects may play an important role in population dynamics, they have been incorporated into simple discrete models of sequential density dependence [2, 7]. We use this approach to investigate potential consequences of carry-over effects in seasonal populations. It is well known that sequential density dependence strongly affects population stability and population size, and carry-over effects can increase the diversity of possible population dynamics, acting either in conjunction or in the opposite direction of regulatory mechanisms [2, 7]. We focus our attention on two relevant phenomena. On the one hand, the possibility of compensatory mortality [1], a paradoxical increase in population size in response to an increasing mortality that has been recently analyzed in detail by Abrams [9] (he coined the term hydra effect that we use here); our results show that carry-over effects have the potential to change population size responses to an increasing mortality, either promoting or neutralizing hydra effects. On the other hand, seasonal density dependence is a potential driver of higher variability [2, 10], and carry-over effects influence these changes in the dynamics of populations. These possible outcomes of seasonality have a special importance when an additional mortality is introduced in the model (e.g., for harvesting, fishing or pest control). In this case, since the size and dynamics of populations are affected by the order of events, harvest timing becomes crucial [2, 3, 11, 12]. Our results emphasize how carry-over effects can change population responses to harvesting, depending in an essential way on harvest timing. Although Ratikainen *et al.* [2] present a thorough review about management implications of density dependence and carry-over effects, as far as we know, this paper provides the first theoretical study about the interplay between carry-over effects and the order of events in a discrete seasonal model.

A thorough discussion on what carry-over effects are or are not can be found in [2, 8]. Here, we follow the definition in page 5 of Harrison *et al.* [8], which is similar to the concept introduced by Norris [7]: *“carry-over effects are events and processes occurring in one season that result in individuals making the transitions between seasons in different levels of condition, consequently affecting individual performance in a subsequent period.”* This definition says that carry-over effects are inter-seasonal effects; on the one hand, the term excludes inter-generational effects (such as maternal effects [4, 13, 14], which are sometimes referred to as developmental effects [8]); on the other hand, the definition of carry-over effects also excludes delayed density dependence, which is defined at the population level as an effect of density in a previous period of population growth [2].

Much of recent work on carry-over effects has been motivated by the study of migratory populations, in which habitat quality during the non-breeding season influences breeding output in the following breeding season. Actually, habitat quality is one of the main processes that drive carry-over effects, but there are other factors such as social status, density, or time constraints (that may reduce fitness if individuals arrive in good condition but with insufficient time to breed) [8]. In the ecological literature there are many examples that evidence carry-over effects in populations; for birds and mammals, we refer to the reviews by Norris and Marra [15] and Harrison *et al.* [8]; for marine species see, e.g., [16].

## Material and Methods

Following [2, 7], we consider a single-species discrete-time population model involving an annual cycle divided into a breeding and a non-breeding season. We will focus our study on the influence of carry-over effects as presented in the introduction, and therefore we do not consider any age or stage structure, assuming a semelparous life history.

The annual cycle begins during the non-breeding season. Denoting by *N*_*b*_(*n*) and *N*_*nb*_(*n*) the population sizes at the end of the breeding and the non-breeding seasons after *n* generations, respectively, the difference equations that govern the population dynamics are

$$\begin{array}{c} N_{b}\left(n\right)=N_{nb}\left(n\right)f\left(N_{nb}\left(n\right)\right), \end{array}$$ (1)

$$\begin{array}{c} N_{nb}\left(n\right)=N_{b}\left(n-1\right)\left(1-d\left(N_{b}\left(n-1\right)\right)\right). \end{array}$$ (2)

Here, *f*(*N*_*nb*_) is the density-dependent per capita breeding output, and *d*(*N*_*b*_) is the mortality rate during the non-breeding season. Since we are interested in the interplay between carry-over effects and compensatory density dependence, we use a Ricker map for the recruitment function, that is, *f*(*x*) = *e*^*r*(1 − *x*/*K*)^, where *r* > 0 and *K* > 0 represent the growth rate and the carrying capacity, respectively. Next, a simple way to introduce carry-over effects in the model consists of a modification in the per capita breeding output [2, 7, 17]. Actually, Betini *et al.* [17] observed in laboratory experiments with *Drosophila* that non-breeding density had a significant effect on per capita breeding output, and that the per capita reproductive success was a linear function of the non-breeding density; thus, they suggested that the carry-over effect could be described replacing *r* by *r* + *aN*_*b*_ in the Ricker map. Using this form for the recruitment, and introducing Eq (2) into Eq (1), we get the following one-dimensional model for the population at the end of the breeding season, accounting for carry-over effects:

$$\begin{array}{c} N_{b}\left(n+1\right)=\left(1-d\left(N_{b}\left(n\right)\right)\right)N_{b}\left(n\right)e^{\left(r+aN_{b}\left(n\right)\right)\left(1-\left(1-d\left(N_{b}\left(n\right)\right)\right)N_{b}\left(n\right)/K\right)}. \end{array}$$ (3)

In this way, *aN*_*b*_(*n*) indicates the magnitude of the carry-over effect, and the sign of *a* determines whether the effects are positive (that is, they increase the growth rate) or negative. As in [17], in our study we only consider admissible values of *N*_*b*_ for which *r* + *aN*_*b*_ is still positive for negative values of *a*.

We carry out a theoretical analysis of the difference Eq (3), with a special attention to the potential impact of carry-over effects on the dynamics. Introducing constant effort harvesting into the model, we also analyze the interplay between carry-over effects and harvesting to infer conclusions regarding management strategies. When we introduce an additional density-independent mortality due to harvesting, there are three different discrete processes in each year: mortality, harvesting and reproduction, so the order of events does matter [2, 3]. If harvesting occurs before mortality and after reproduction, then the sequence of events is

$$\begin{array}{c} \text{Harvesting}⟶\text{Mortality}⟶\text{Reproduction.} \end{array}$$

Denoting by *h* the strength of the harvesting effort, and introducing a new variable *N*_*h*_ for the population size after harvesting, we get

$$\begin{array}{c} N_{h}\left(n\right)=\left(1-h\right)N_{b}\left(n\right)\;;\;N_{nb}\left(n\right)=N_{h}\left(n-1\right)\left(1-d\left(N_{h}\left(n-1\right)\right)\right), \end{array}$$

so, replacing these expressions in Eq (1) provides the following model, that we will refer to as HMR:

$$\begin{array}{c} N_{b}\left(n+1\right)=\left(1-h\right)\left(1-d\left(\left(1-h\right)N_{b}\left(n\right)\right)\right)N_{b}\left(n\right)e^{\left(r+aN_{b}\left(n\right)\right)\left(1-\left(1-h\right)\left(1-d\left(\left(1-h\right)N_{b}\left(n\right)\right)\right)N_{b}\left(n\right)/K\right)}. \end{array}$$ (4)

If, on the contrary, harvesting occurs after mortality and before reproduction, we have the sequence

$$\begin{array}{c} \text{Mortality}⟶\text{Harvesting}⟶\text{Reproduction.} \end{array}$$

In this case, *N*_*h*_(*n*) = (1 − *h*)*N*_*nb*_(*n*), and we arrive at the MHR model

$$\begin{array}{c} N_{b}\left(n+1\right)=\left(1-h\right)\left(1-d\left(N_{b}\left(n\right)\right)\right)N_{b}\left(n\right)e^{\left(r+aN_{b}\left(n\right)\right)\left(1-\left(1-h\right)\left(1-d\left(N_{b}\left(n\right)\right)\right)N_{b}\left(n\right)/K\right)}. \end{array}$$ (5)

Note that Models (4) and (5) assume that population is measured after reproduction. With this convention, HMR and MHR are the only possible relative positions for the three involved discrete events.

## Results

### Isolating the impact of carry-over effects on population abundance

Since carry-over effects are introduced in Eq (3) as a modification of the population growth rate parameter *r*, it is expected that negative carry-over effects in a generation lead to a lower population abundance that could have been attained without carry-over effects, and vice versa. These responses have been observed in previous works [2, 7, 17], and we obtain results in this direction. For example, for stable populations with density-independent mortality, we prove in Proposition 1 in the Appendix that the size of the equilibrium decreases with negative carry-over effects. However, for unstable populations the contribution of carry-over effects can be more subtle. We use a simple mechanism to isolate the contribution of carry-over effects: for a given value of the carry-over strength parameter *a*, let {*N*_*b*_(*n*)} be the sequence obtained from Eq (3) starting at an initial condition *N*_*b*_(0). For each *n*, we define

$$\begin{array}{c} O\left(n\right)=\left(1-d\left(N_{b}\left(n-1\right)\right)\right)N_{b}\left(n-1\right)e^{(r\left(1-\left(1-d\left(N_{b}\left(n-1\right)\right)\right)N_{b}\left(n-1\right)/K\right)}. \end{array}$$ (6)

We notice that *O*(*n*) is the population abundance at the end of the *n*-th breeding season when *N*_*b*_(*n* − 1) is the population density at the end of the previous breeding season and carry-over effects are ignored in the *n*-th annual cycle. Therefore, the contribution of the carry-over effects on population size at the *n*-th generation could be measured as

$$\begin{array}{c} C\left(n\right)=N_{b}\left(n\right)-O\left(n\right). \end{array}$$ (7)

**Fig 1:**
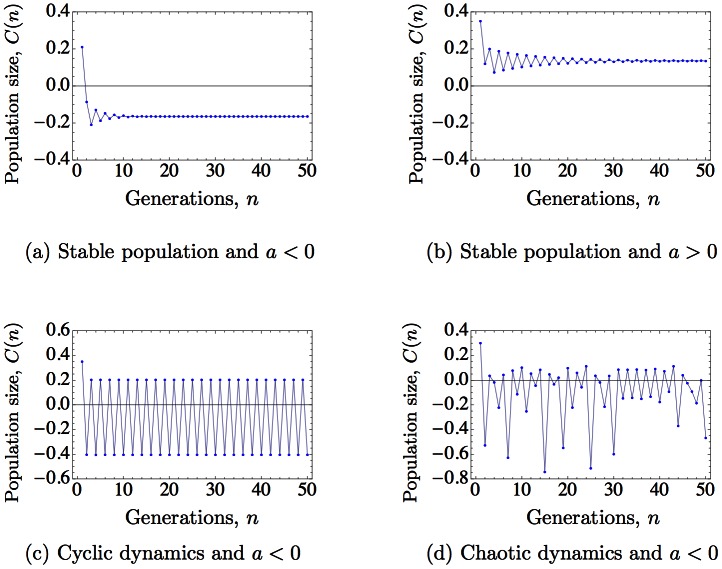
Contribution *C*(*n*) of the carry-over effects on population abundance during 50 generations, as defined by Eq (7). Under simple dynamics ((*a*)–(*b*)), contributions tend to be constant in the long term (with negative contribution for negative carry-over effects and vice versa). In an oscillatory or chaotic regime ((*c*)–(*d*)), the contribution of carry-over effects is also oscillating, showing both positive and negative contributions. We use Eqs (3), (6) and (7) with constant *d*, *K* = 1, and different parameters. (*a*): *r* = 2.6, *a* = −0.4, *d* = 0.6; (*b*): *r* = 2, *a* = 0.4, *d* = 0.6; (*c*): *r* = 3.7, *a* = −0.4, *d* = 0.5; (*d*): *r* = 4.2, *a* = −0.4, *d* = 0.6.

In stable populations, we observe that positive carry-over effects indeed promote population increase, and negative carry-over effects promote population decline. Moreover, after some transients, the impact of carry-over effects on population abundance tends to be constant (Fig 1(a) and 1(b)). However, if the population exhibits sustained oscillations or chaotic dynamics, the impact of carry-over effects, as measured by Eq (7), can be oscillatory, that is, regardless the sign of *a*, in some generations carry-over effects lead to higher population abundance and in others we observe the opposite response (Fig 1(c) and 1(d)).

### Carry-over effects and the hydra effect

For a better understanding of Model (3), we notice that, in the absence of carry-over effects (that is, *a* = 0 in Eq (3)) and assuming a constant mortality rate *d*, the model reduces to

$$\begin{array}{c} N_{b}\left(n+1\right)=\left(1-d\right)N_{b}\left(n\right)f\left(\left(1-d\right)N_{b}\left(n\right)\right), \end{array}$$ (8)

where *f*(*x*) = *e*^*r*(1 − *x*/*K*)^. Eq (8) is well studied (see, e.g., Eq (4) in [9] and Eq (8) in [18]). In particular, this model can exhibit a hydra effect [9].

**Fig 2:**
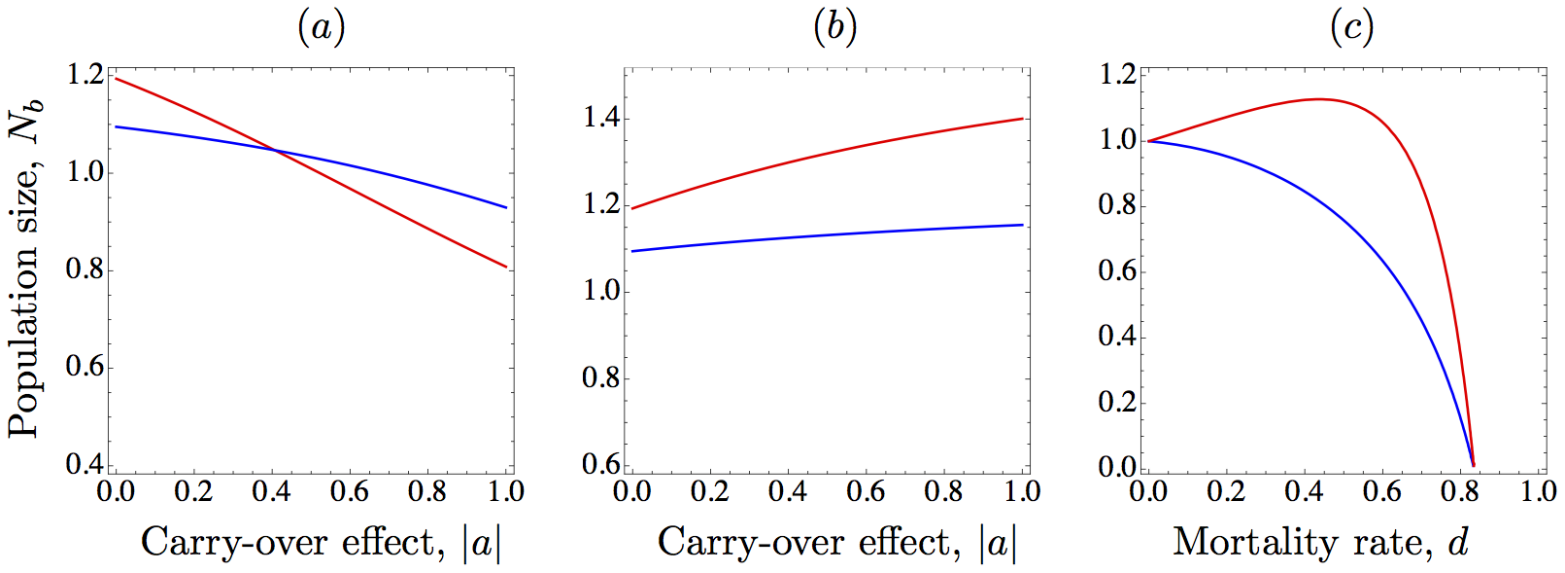
Population size response to increasing mortality in the presence of carry-over effects. In the three figures, the equilibrium is asymptotically stable and we show its variation using the intensity of the carry-over effect ((*a*) and (*b*)), or the per capita mortality rate (*c*) as the bifurcation parameters. We use Eq (3) with constant *d*, *K* = 1, and *r* = 1.8. (*a*) Ignoring carry-over effects, an increase of the mortality rate from *d* = 0.2 (blue) to *d* = 0.4 (red) results in an increase of population size; this compensatory effect is prevented as the intensity of negative carry-over effects is increased. (*b*) The same parameters as in (*a*), but with positive carry-over effects; contrary to what happens for negative carry-over effects, an increasing intensity tends to magnify compensatory effects; (*c*) using the mortality rate as the bifurcation parameter, we show how the hydra effect observed for Eq (3) with a negative intensity of the carry-over *a* = −0.2 (red line) is no longer observed increasing the intensity of carry-over effects to *a* = −0.9 (blue line).

An interesting outcome of negative carry-over effects is that, when they are strong enough, they tend to neutralize the paradoxical hydra effect (see Fig 2). This phenomenon can be interpreted as a natural consequence of the reduction in the growth rate because the hydra effect requires moderated values of *r* to occur in Model (8) [9, 19, 20].

### Carry-over effects and stability

First, we notice that if we consider a model with density-independent carry-over effects (which means that the growth rate parameter *r* is replaced by *r* + *a* instead of *r* + *aN*_*b*_(*n*) in Eq (3)), increasing the strength of negative carry-over effects results in a standard stabilization of the positive equilibrium by a cascade of periodic halving bifurcations, an effect that has been already studied in the context of harvesting models for Eq (8) [20]. Accordingly, positive carry-over effects tend to increase complexity, generating a route to chaos as the strength of carry-over effects increases.

The destabilizing impact of positive density-dependent carry-over effects is also observed in numerical simulations with Eq (3). We now focus on negative density-dependent carry-over effects and consider two different forms for the mortality rate during the non-breeding season.

**Fig 3:**
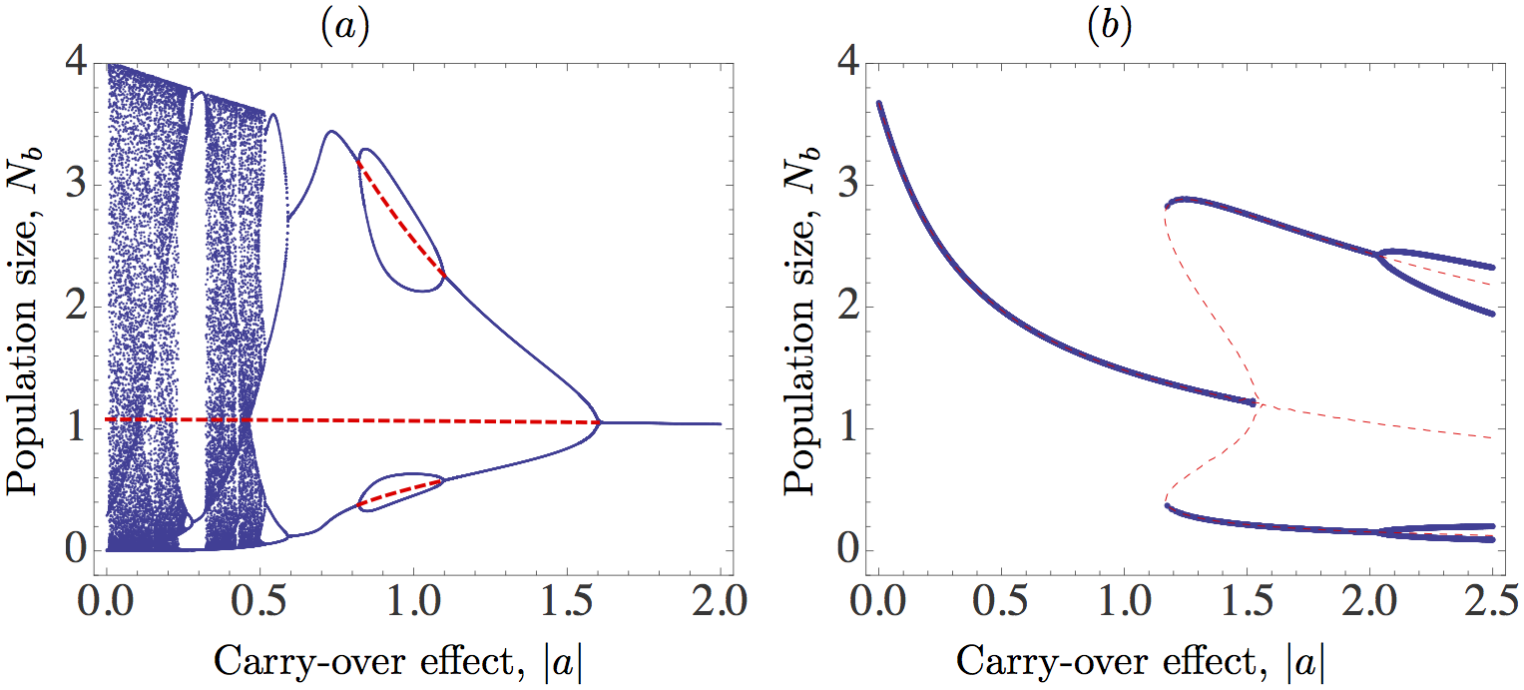
Bifurcation diagrams showing the interplay between negative carry-over effects and stability. In (*a*)–(*b*), the long-term behavior of the solutions is plotted as the strength of the carry-over effects |*a*| is increased; red dashed lines correspond to the unstable equilibrium and some unstable 2-cycles. (*a*) Density-independent mortality using Eq (3) with *K* = 1, *r* = 3.7, *d*(*N*_*b*_) ≡ *d* = 0.1; carry-over effects are stabilizing but the route from chaos to stability is not the usual one since stability switches can appear once the periodic regime reaches a period-two solution. (*b*) We use Eq (3) with density-dependent mortality *d*(*N*_*b*_) = 1 − exp(−*αN*_*b*_), *K* = 1, *r* = 3.65, and *α* = 0.8. Increasing the strength of carry-over effects can destabilize the equilibrium and give rise to the coexistence of two stable attractors for |*a*|∈ [1.16, 1.54]: a positive equilibrium and a 2-cycle.

- When the mortality rate *d* is constant, our analytical results in the Appendix establish that a stable population cannot be destabilized in response to an increasing strength of carry-over effects, and our bifurcation diagram in Fig 3(a) shows that a cyclic or chaotic population is stabilized as the strength of carry-over effects increases. However, the stabilizing effect can be more involved than the usual period-halving route if the values of the growth rate *r* are moderated: stability switches (period-doubling reversals) can appear, showing the so-called bubbling effect in the bifurcation diagram (Fig 3(a)). This phenomenon has been observed, among others, in models involving immigration [21], and in harvesting models for populations with adult survivorship [18, 22, 23].
- When both mortality and breeding are density-dependent, their interplay can have destabilizing effects on population size [10]. It is natural to expect that in this case carry-over effects can destabilize. Indeed, we deal with a density-dependent survivorship rate *s*(*N*_*b*_(*n*)) = 1 − *d*(*N*_*b*_(*n*)) = exp(−*αN*_*b*_(*n*)). Taking the strength of the carry-over effect |*a*| as a bifurcation parameter and choosing *r* = 3.65, *α* = 0.8, a population which is globally stable without carry-over effects first exhibits bistability and then is destabilized in response to an increase in |*a*| (Fig 3(b)).

### Carry-over effects and harvesting

The interplay between carry-over effects and sequential density dependence has important implications for the management of populations affected by negative effects in the non-breeding season, such as habitat loss or lack of resources [2]. As we have shown in the previous subsections, negative carry-over effects can promote population fluctuations, which might lead to extinction of endangered populations by stochastic perturbations. Thus, it is crucial to examine how harvesting interacts with the other events involved in the model. Since in a discrete model with more than two events the relative order of events does matter, we have considered Models (4) and (5), corresponding to harvesting-mortality-reproduction (HMR) and mortality-harvesting-reproduction (MHR), respectively. We fix the mortality rate *d* and parameters *K* and *r* to explore how changes in harvesting effort affect population dynamics for different strength values of the carry-over effects.

**Fig 4:**
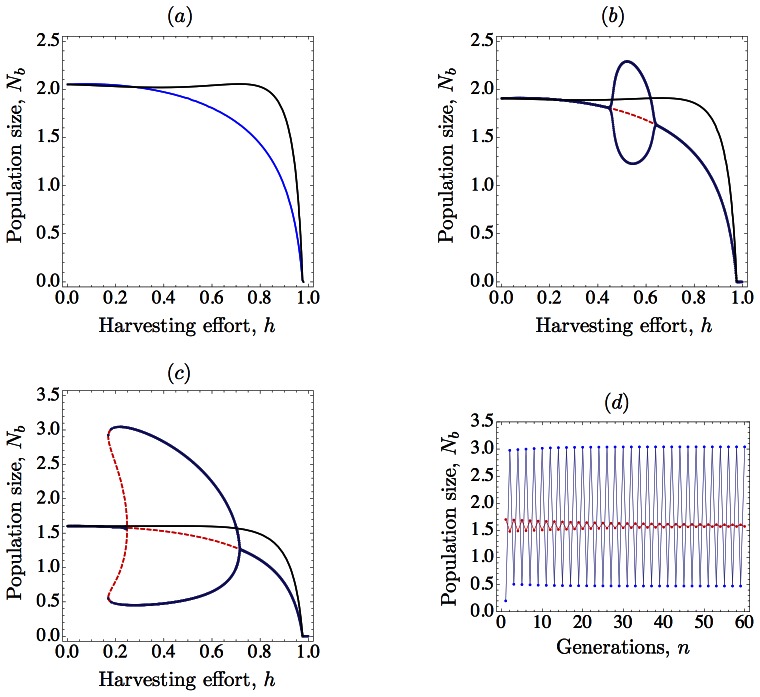
Consequences of harvest timing under negative carry-over effects. In (a)-(c), the long-term behavior of the solutions is plotted as harvesting effort is increased both for harvesting after mortality (blue, which corresponds to Eq (5) in the text) and harvesting before mortality (black, which corresponds to Eq (4) in the text); red dashed lines correspond to the unstable equilibrium in (b) and (c), and an unstable 2-cycle in (*c*). In all cases, harvesting before mortality gives higher average population number. We use Eqs (4) and (5) with *K* = 1, *r* = 3.75, and density-dependent mortality *d*(*x*) = 1 − exp(−*αx*) with *α* = 0.8. As the intensity of a negative carry-over effect is increased, we observe an interesting transition in the bifurcation diagram for harvesting after mortality. (*a*) For *a* = −0.5, increasing harvesting does not destabilize the equilibrium. (*b*) For *a* = −0.6, there is a stability switch (*bubbling*). (*c*) For *a* = −0.9, increasing harvesting destabilizes the equilibrium and give rise to the coexistence of two stable attractors: a positive equilibrium and a 2-cycle. We show this bistability in (*d*) for *h* = 0.2 and initial conditions *N*(0) = 1.7 (red) and *N*(0) = 0.2 (blue). Remarkably, harvesting before mortality does not destabilize the equilibrium in any of the cases (a)-(c).

As for the possibility of compensatory mortality (hydra effect), our results are in agreement with previous studies (e.g., [1, 2, 11]). Namely, a model with harvesting prior to mortality (HMR) gives a higher average population number than the corresponding model with harvesting after mortality (MHR), and, accordingly, hydra effects are more likely to happen in (HMR) models; however, these effects are less severe for stronger negative carry-over effects (see Fig 4).

Perhaps the most interesting outcome of our results is that harvesting after mortality leads to a higher variability in population abundance, and this effect is stronger when the intensity of carry-over effects increases. In this sense, our results emphasize the relevance of harvest timing when carry-over effects enter into the game. Indeed, Fig 4 shows that, for the same parameter values, population dynamics is hardly affected by carry-over effects if harvesting precedes mortality (HMR), while the bifurcation diagrams for the MHR model display a transition in the dynamics from a globally stable equilibrium to instability and then to bistability as the strength of carry-over effects increases.

## Discussion

In seasonal populations, there are multiple factors that affect life-histories, especially when different sequential density-dependent mechanisms operate [2]. Although there is a huge literature dealing with life-history population responses to seasonal changes and individual-level performances (e.g., [2, 4–6, 13, 24–26]), these models usually involve delayed life-history effects or structured populations. Theoretical and field work shows that, for many species, processes during the non-breeding period influence vital rates in a subsequent period of the same annual cycle [7, 17, 27]. We have carried out a theoretical study of a discrete semelparous population model with an annual cycle divided into a breeding and a non-breeding season; we assume two events (mortality and breeding) and carry-over effects coming from the non-breeding period and affecting breeding performance through a density-dependent adjustment of the growth rate parameter within the same annual cycle. This model has been tested in a laboratory experiment [17], but little is known about its potential dynamic outcomes. On the one hand, we have investigated the influence of carry-over effects on population size, focusing on two important aspects: compensatory mortality and population variability. On the other hand, to understand the potential consequences of carry-over effects for management, we have introduced a third event in the form of constant effort harvesting. Since there are three discrete events, their relative order becomes crucial. Depending whether harvesting occurs prior to mortality or after it, our results show that carry-over effects can either have very little effect in the dynamics when harvesting pressure is increased or, on the contrary, they can induce dramatic changes in population stability.

A prevailing principle in management theory for the sustainability of a population is that harvest mortality should take place before reproduction. Thereby the population can compensate the removal of individuals by increasing survival and productivity [2, 11, 12]. It is well known that sequential density dependence opens the possibility for compensatory mortality [1], that is, population size can increase as a response to greater mortality. This paradoxical effect is known as hydra effect, and it is typical for discrete models when mortality precedes an overcompensatory density dependence, as in the Ricker model [9]. In agreement with Norris [7], our model predicts that negative carry-over effects tend to neutralize this effect because they reduce intrinsic population growth rates. Since many species undergo negative carry-over effects, this remark might be an argument to explain why there is little evidence of hydra effects in nature, especially if stage-structure is not considered [9, 28].

The potential destabilizing consequences of life-history effects, especially if they are delayed, is well known [4, 13, 24, 25]. Also, although it has been usually assumed that harvesting is stabilizing [3], it is now recognized that it can magnify fluctuations in population abundance, especially in age-structured populations [22, 23, 29, 30]. Recent studies show that size-selective fishing induces life-history changes that can destabilize ecosystems [31, 32].

In this direction, a first result of our work is that negative carry-over effects tend to be stabilizing when the mortality rate is constant, but a stable population can be destabilized as the strength of carry-over effects increases if mortality is density-dependent. Second, the interplay of moderate values of harvesting effort and carry-over effects can be seen as a new mechanism why harvested populations can fluctuate more than unharvested stocks; this effect seems to be more likely observable if harvesting occurs after mortality and before breeding, which highlights the importance of harvest timing for the management of populations [2, 11, 12, 33].

In summary, the results of this paper aim to be a new step towards the theoretical understanding of the biological consequences of carry-over effects in management. Considering harvesting and carry-over effects in a simple seasonal model reveals how potential dramatic changes may appear in populations as a response to changes in parameter values (harvesting effort, strength of carry-over effects, mortality rate).

## Appendix

The aim of this section is to discuss how negative carry-over effects contribute to stabilize population oscillations in the case of density-independent survivorship during the non-breeding period. To do that, we show that, under some mild conditions, Eq (3) has a nontrivial equilibrium that is attracting for a range of parameters wider than the corresponding equilibrium of the same equation ignoring carry-over effects (*a* = 0). It is not restrictive to assume that *K* = 1, since if does not affect the stability properties of the equilibria. Thus, denoting *x*_*n*_ = *N*_*b*_(*n*) for simplicity, Eq (3) becomes

$$\begin{array}{c} x_{n+1}=\alpha x_{n}e^{\left(r+ax_{n}\right)\left(1-\alpha x_{n}\right)}:=G\left(x_{n}\right), \end{array}$$ (9)

with *r* > 0, *a* ≤ 0, and *α* = 1 − *d* ∈ [0, 1). The limit case *a* = 0 corresponds to the model ignoring carry-over effects

$$\begin{array}{c} x_{n+1}=\alpha x_{n}e^{r(1-\alpha x_{n})}:=F\left(x_{n}\right). \end{array}$$ (10)

A straightforward computation implies that a positive equilibrium of Eq (10) exists if and only if *r* + log *α* > 0. Such a point is given by

$$\begin{array}{c} \bar{x}=\frac{r+\log\alpha }{\alpha r}, \end{array}$$

and satisfies that

$$\begin{array}{c} F^{\prime }\left(\bar{x}\right)=1-\left(r+\log\alpha \right). \end{array}$$

On the other hand, for *a* < 0 the nontrivial equilibria of Eq (9) are given by the roots of

$$\begin{array}{c} \log\alpha +(r+ax)(1-\alpha x)=0. \end{array}$$ (11)

If *r* + log *α* > 0, there are two positive roots, namely

$$\begin{array}{c} x_{*}=\frac{\left(r\alpha -a\right)-\sqrt{{\left(r\alpha +a\right)}^{2}+4a\alpha \log\alpha }}{2|a|\alpha }\;,\;x_{**}=\frac{\left(r\alpha -a\right)+\sqrt{{\left(r\alpha +a\right)}^{2}+4a\alpha \log\alpha }}{2|a|\alpha }. \end{array}$$

If *r* + log *α* ≤ 0, then Eq (11) has a unique positive solution x_**_ and all solutions of Eq (9) with initial condition in [0, x_**_) tend to zero.

Since x_**_ is always unstable, we restrict our attention to the stability properties of x_*_, with multiplier $G^{\prime }\left(x_{*}\right)=1-x_{*}\left(\sqrt{{\left(r\alpha +a\right)}^{2}+4a\alpha \log\alpha }\right).$ It is not difficult to find sufficient conditions to ensure that *G* maps the interval *J* = [0, x_**_) into itself; we assume that this property holds, and take only admissible initial conditions *x*_0_ ∈ *J*.

The following result establishes that, under density-independent mortality, negative carry-over effects have a stabilizing effect and tend to decrease population size at equilibrium.

**Proposition 1**. *Assume that* *r* + log *α* > 0. *Then* $x_{*}<\bar{x}$ *and*

$$\begin{array}{c} F^{\prime }\left(\bar{x}\right)<G^{\prime }\left(x_{*}\right)<1. \end{array}$$ (12)

**Proof**. It is straightforward to verify that $x_{*}<\bar{x}$. Next, to prove Eq (12), we have to check that

$$\begin{array}{c} x_{*}\left(\sqrt{{\left(r\alpha +a\right)}^{2}+4a\alpha \log\alpha }\right)<r+\log\alpha . \end{array}$$

This inequality is equivalent to

$$\begin{array}{c} -2a\alpha \left(r+\log\alpha \right)>\left(-a+r\alpha \right)\sqrt{{\left(r\alpha +a\right)}^{2}+4a\alpha \log\alpha }-{\left(r\alpha +a\right)}^{2}-4a\alpha \log\alpha , \end{array}$$

which reduces to

$$\begin{array}{c} a^{2}+r^{2}\alpha^{2}+2a\alpha \log\alpha >\left(-a+r\alpha \right)\sqrt{{\left(r\alpha +a\right)}^{2}+4a\alpha \log\alpha }. \end{array}$$

Getting rid of the square root and simplifying, we obtain that Eq (12) is equivalent to the inequality

$$\begin{array}{c} 4a^{2}\alpha^{2}{\left(r-\log\alpha \right)}^{2}>0, \end{array}$$

which obviously holds.
